# *Aloe vera* Induced Biomimetic Assemblage of Nucleobase into Nanosized Particles

**DOI:** 10.1371/journal.pone.0032049

**Published:** 2012-03-05

**Authors:** Arun Chauhan, Swaleha Zubair, Asif Sherwani, Mohammad Owais

**Affiliations:** 1 Interdisciplinary Biotechnology Unit, Aligarh Muslim University, Aligarh, Uttar Pradesh, India; 2 Women's College, Aligarh Muslim University, Aligarh, Uttar Pradesh, India; Northeastern University, United States of America

## Abstract

**Aim:**

Biomimetic nano-assembly formation offers a convenient and bio friendly approach to fabricate complex structures from simple components with sub-nanometer precision. Recently, biomimetic (employing microorganism/plants) synthesis of metal and inorganic materials nano-particles has emerged as a simple and viable strategy. In the present study, we have extended biological synthesis of nano-particles to organic molecules, namely the anticancer agent 5-fluorouracil (5-FU), using *Aloe vera* leaf extract.

**Methodology:**

The 5-FU nano- particles synthesized by using *Aloe vera* leaf extract were characterized by UV, FT-IR and fluorescence spectroscopic techniques. The size and shape of the synthesized nanoparticles were determined by TEM, while crystalline nature of 5-FU particles was established by X-ray diffraction study. The cytotoxic effects of 5-FU nanoparticles were assessed against HT-29 and Caco-2 (human adenocarcinoma colorectal) cell lines.

**Results:**

Transmission electron microscopy and atomic force microscopic techniques confirmed nano-size of the synthesized particles. Importantly, the nano-assembled 5-FU retained its anticancer action against various cancerous cell lines.

**Conclusion:**

In the present study, we have explored the potential of biomimetic synthesis of nanoparticles employing organic molecules with the hope that such developments will be helpful to introduce novel nano-particle formulations that will not only be more effective but would also be devoid of nano-particle associated putative toxicity constraints.

## Introduction

Nanosized particles acquire unusual but beneficial properties which may find industrial applications in biotechnology as well as other fields with equal propensity [1], [2]. The specific size of nano-particles is considered to be of great importance for their application as cell marker in bio-sensing, bio-imaging and targeted drug delivery *etc* [1]–[4]. When used as delivery vehicle, the nano-size range of the particles of therapeutic importance not only makes them suitable for development of systemic, oral, pulmonary, trans-dermal and other routes of delivery but also helps in increasing bioavailability, protection of bioactivity, stability and eventually in targeted delivery of the drug of interest. In fact, the nano-structured drug particles have the ability to permeate cells and connective tissues and thus can be targeted efficiently to the desired biological site without clogging the capillaries. It is well appreciated that size of the formulation regulates its uptake by cells that eventually results in greater accumulation of associated drug at the desired site. Once internalized, the drug formulation can be retained by diseased/infected tissues for longer time thereby increasing bioavailability and eventually the efficacy of the drug [5].

Biomimetic synthesis of nano-particles is quite common in both single as well as multi cell organisms. In multi-cellular organisms, synthesis of hard tissues such as shells, spicules, teeth and bones *etc* takes place through a co-ordinated process, for example, bone formation is mediated by osteoblasts while dentinoblasts are responsible for dentin formation [6], [7]. In most of such naturally occurring structures the particles consist of highly intricate and ordered architectures with well defined shapes. Interestingly, the synthesized particles are formed within a certain size range, orientation and geometrical symmetry. Various structures, thus, formed are highly organized at molecular, nano and macroscales.

Both bacteria and plant extracts have been lately found to possess unique properties of synthesizing metal based nano-particles. In spite of several efforts to dissect various possible modes of biomimetic synthesis, it is still not clear that what specific components are present in these extracts that lead to the formation of nano-assemblies [8]. It is speculated that cytosolic as well as secretory components of living cells might offer a template that facilitates the formation of nano-assembly in an aqueous environment [9]–[12]. Earlier work on biomimetic synthesis of nano-particles suggested that organic molecules present in the cytosol of the microbes or plant cells facilitate transport and collection of materials leading to the formation of self and co-assembled nano-particles with uniform consistency [9], [10]. Leaf extracts from *Aloe vera*, *Azardirachta indica*, *Cymbopogon flexuosus* have been used in the synthesis of gold, silver, cadmium and bismuth nano-particles [9], [11], [12]. Taking a lead from the earlier work on biomimetic synthesis of metal and inorganic material nano-particles, we have determined the effect of *Aloe vera* leaf extract on organic molecules. To this end we have used the nucleobase analog, 5-fluorouracil (5-FU) as a model compound. 5-FU is one of the most potent anti-cancer agents used in the treatment of colorectal, head and neck, pancreatic and breast carcinoma. It is a pro-drug that requires conversion to 5-fluorodeoxyuridine monophosphate and 5-fluorodeoxyuridine triphosphate in cancer cells [13], [14]. The drug is also available in the form of ointment based formulation that is widely used for topical application [15].

In the present work, we describe a simple method to synthesize 5-FU nano particles using *Aloe vera* leaf extract. The synthesized 5-FU nano-particles were characterized by various microscopic as well as spectrophotometric techniques. Next, to see whether 5-FU nano-particles still retain anti-cancer activity, a cell cytotoxicity study was done using HT-29 and Caco-2 (human adenocarcinoma colorectal) cell lines.

## Results

The colorless mucilaginous gel of *Aloe vera* leaves is widely used as anti-bacterial, neutraceutical, anti-inflammatory and immunomodulating agent [16]. Recently, it has been reported to help in the bioconversion of metal as well as inorganic materials to nano-assemblies [12]. We wondered whether the potential of *Aloe vera* leaf extract to form inorganic material nano-particles could be extended to formation of organic molecule nano-particles as well. Surprisingly, it was found that incubation of 5-FU with *Aloe vera* leaf extract ensues in the bio-formation of 5-FU nano-particles.

The biomimetic potential of *Aloe vera* leaf extract was first established by evaluating its ability to convert chloroauric acid (HAuCl_4_) to nano-sized gold particles [12]. Incubation of *Aloe vera* leaf extract with aqueous HAuCl_4_ solution led to the appearance of a mauve red color in solution after about 12 h of incubation, indicating the formation of gold nano-particles (data not shown). The gold nano-particle synthesis was further confirmed by visible absorption spectrum of the solution that shows characteristic surface plasmon resonance (SPR) band of gold nano-particles centered at 560 nm (Figure S1). In the next phase of study, we used the same leaf extract for preparation of 5-FU nano-particles.

### UV spectral analysis

**Figure 1:**
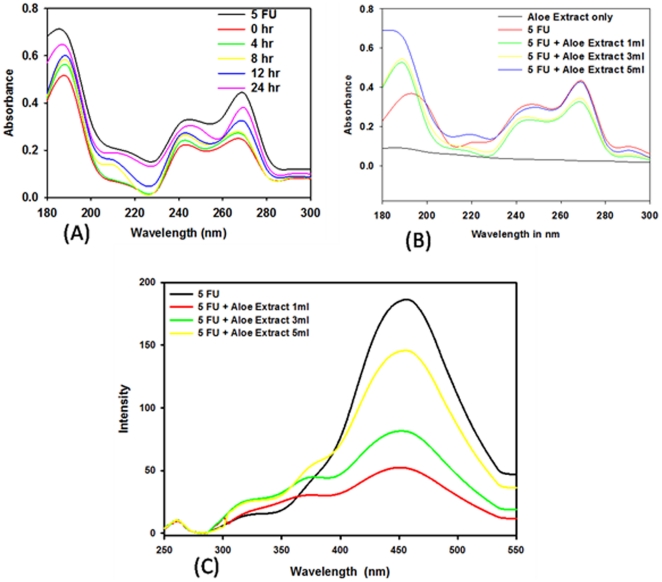
Spectrophotometric analysis of 5-FU nano-particles. (**A**) Time kinetic effect of Aloe vera leaf extract mediated synthesis of 5-FU nano-particles employing UV Spectrophotometric analysis. The 5-FU solution was incubated with *Aloe vera* extract (5 ml of 10^−3^ M 5-FU and 5 ml *Aloe vera* extract; final volume of reaction mixture 10 ml) for specified time period (0–24 h). The incubation mixture was scanned in UV range to analyze characteristic peaks. The spectrum shown at zero time point actually corresponds to 5-FU nanoparticles obtained instantly just after initial mixing of free 5-FU with *Aloe vera* leaf extract. (**B**) Concentration dependent kinetics of Aloe vera leaf extract mediated synthesis of 5-FU nano-particles as revealed by UV Spectrophotometric analysis. UV spectra of 5-FU nano-particles synthesized using increasing amounts of *Aloe vera* extract. (**C**) Emission spectra of 5-FU nano-particles synthesized using *Aloe vera* extract. The 5-FU solution (10^−3^ M) was incubated with increasing volume of *Aloe vera* extract and scanned employing spectrofluorimeter. The excitation wave length was 260 nm.

The UV absorption spectra were recorded both for aqueous suspension of 5-FU nano-particles as well as its free form (Figure 1A). The free drug absorbs maximally at 271 nm. Mixing of 5-FU with *Aloe vera* leaf extract resulted in instant quenching of 5-FU absorbance. For the sake of simplicity, the observed absorption spectrum was annotated as zero time point observation (Figure 1A). Interestingly, longer incubation results in resurgence of absorbance with significant increase in intensity of various characteristic peaks in a time dependent manner. Following abrupt quenching, the resurrection of original peaks with time indicates that synthesized 5-FU nano-particles too absorb specifically at the same wavelengths. For example, the significant higher absorption at ∼245 nm and ∼192 nm suggests that C = O chromophore along with heteroatom (-N) containing the conjugated pyrimidine ring remains intact in both free as well as nano-assembled drug molecules (Figure 1A). The significant blue shift in the region at 272 nm suggests that chromophores are actively involved in non-covalent bonding of nucleobase and eventually participate in formation of nano-particles.

It has been suggested that microenvironment of chromophoric groups of a chemical compound affects the Sπ life-span mainly by tuning the relative energy of the Sπ state and the closely lying Sn dark state [17]. The stability of π-π* transition (in the case of 5-FU; it is the transfer of an electron from the lone pair of oxygen or nitrogen atom towards the more diffuse π* orbital) increases both with the polarity and hydrogen bonding ability of 5-FU with the external milieu. Further, it implies that with such microenvironment, the dynamics of Sπ is not influenced by Sn. Moreover, since the nano-particle framework is providing relatively less polar environment to the entangled drug molecules when compared to that of free form of the drug, it eventually results in broadening of the absorption spectrum. It seems that in nano-particle assembly, the external milieu of an individual 5-FU molecule modulates both π-π* as well as n-π* transitions [17], [18].

The concentration dependent kinetics of nano-particle synthesis is more intriguing. At a fixed time point (12 h), the absorbance was found to decrease upon addition of *Aloe vera* leaf extract (1 ml *Aloe* extract; final volume of reaction mixture 10 ml) to 5 ml of 10^−3^ M 5-FU solution (Figure 1B). *Aloe vera* mediated transformation of drug to nano-size assembly results in considerable decrease in the absorption peaks. However, mixing of drug with relatively larger ratio of *Aloe vera* extract (3 ml *Aloe* extract and 5 ml of 10^−3^ M 5-FU solution in final volume of the 10 ml) brings about an increase in absorbance. Addition of more *Aloe vera* extract to 5-FU (5 ml extract and 5 ml of 10^−3^ M 5-FU solution in final volume of 10 ml) ensued in additional increase in peak intensity bringing the curve nearer to that of free form of the drug. This clearly suggests that higher concentration of the active components of *Aloe vera* leaf extract facilitates ready formation of drug nano-particles without changing the chemical structure of the nucleobase.

### Fluorescence spectra analysis

The nucleobase 5-FU is highly fluorescent, possessing characteristic excitation and emission wavelengths [17]–[19]. Addition of *Aloe vera* leaf extract to 5-FU resulted in quenching of fluorescence intensity (Figure 1C). The observed decrease in relative fluorescence intensity of 5-FU does not seem to be due to the interaction of fluorophore moieties of the compound with *Aloe vera* leaf extract components that come in contact to each other during process of nano-particle formation. We also ruled out self quenching, an intrinsic property exhibited by certain fluorophores at high concentrations to be the probable cause of decrease in fluorescence intensity of nanoparticles [20], [21]. Rather, the observed quenching can be attributed to the active involvement of fluorophore in interaction between various 5-FU molecules constituting the nano-particles. In-fact, the fluorescence spectra of free 5-FU suggest that at least 4 different fluorophore species are responsible for fluorescence behavior of 5-FU. Among these C = O and –NH- are the main contributors. The fluorescence emissions are also contributed from vibrational modes involving C_5_–C_6_ stretching as well as out of plane motion of carbonyl groups.

The noticeable feature concerns the relative energy of the first absorption band (S_O_→S_1_) which corresponds to a π→π* transition. The energy trends can be correlated well with the shape of the orbits involved in the S_O_→S_1_ transition. The fluoro group at C_5_ position gives an anti-bonding contribution to the frontier orbitals, thereby making them less stable. It can be inferred that when substituent at C_5_ position (*cf*. -fluoro) gives rise to hyper conjugation effect, the S→S_1_ transition is shifted towards lower energy as compared to uracil [17], [18]. On the contrary, an increase in electron density on the C_4_ carbonyl group is likely to be responsible for the Sπ stabilization. During nano-assembly formation, C = O as well as –NH- groups might be facilitating the stacking of 5-FU molecules and that in-turn leads to abrupt quenching of nano-formulation. As more than one fluorophore species are involved in fluorescence event, quenching is more obvious for the fluorophores which are directly involved in nano-particle assembly formation.

In aqueous environment, 5-FU shows hydrogen bonding that involves formation of H_2_O shell around the periphery of each molecule. Upon nano-particle formation, 5-FU molecules adopt a three dimensional frame work where inter-molecular hydrogen bonds provide sufficient stability to the particles. The observation that 5-FU nano-particles retain fluorescence characteristic of the parent nucleobase again led us to believe that *Aloe vera* extract helps in assemblage of nano-particles of 5-FU. Further, in a given 5-FU nano-particle assembly, various molecules seem to be oriented in the fixed position by non-covalent linkages as there is no significant change in the fluorescence characteristic of 5-FU parent molecule. It has been suggested that Sπ arises mainly from the HOMO→LUMO excitation with π/π* character, whereas Sn has predominant HOMO-1→LUMO character (n/π*) [17], [18]. Incidentally, various moieties that participate in hydrogen bonding are responsible for n/π* and π/π* electronic character of 5-FU as well.

The observed change in fluorescence pattern can also be correlated with shape and size of the particles as in the next section which deals with transmission electron microscopic studies; we clearly observed an effect of increase in *Aloe vera* leaf extract concentration on size of the synthesized particles. Less amount of *Aloe vera* leaf extract results in synthesis of relatively large sized nano-particles, which show less fluorescence intensity due to quenching. Higher ratio of leaf extract to drug solution leads to synthesis of relatively small sized nano-particles, eventually resulting in lesser quenching and higher fluorescence intensity. The observed quenching in the relative fluorescence can be attributed to the assemblage of 5-FU molecules in nano-sized particles.

The fluorescence spectrum shown in Figure 1C, clearly demonstrates that left shoulders are significantly blue shifted. This trend can be correlated to the shape of the frontier orbitals of 5-FU and can also be attributed to the increase of dipole moment upon electronic excitation [17], [18]. The observed blue shift of 5-FU nano-particle indicates that fluorophores are experiencing relatively less polar environment. As explained for the observed quenching of relative fluorescence, this situation is possible when 5-FU molecules are stacked upon each other thereby leading to the formation of compact particulate assembly. Further, the observed change in pattern of fluorescence intensity suggests a role of intermolecular hydrogen bonds in modulating the interplay between dark and bright states and consequently, the excited state life time of 5-FU. The fluorescence data, obviously related to the complexity of the excited state decay mechanism, suggest that several effects are operative in observed fluorescence quenching.

### TEM analysis of synthesized nano-particles

**Figure 2:**
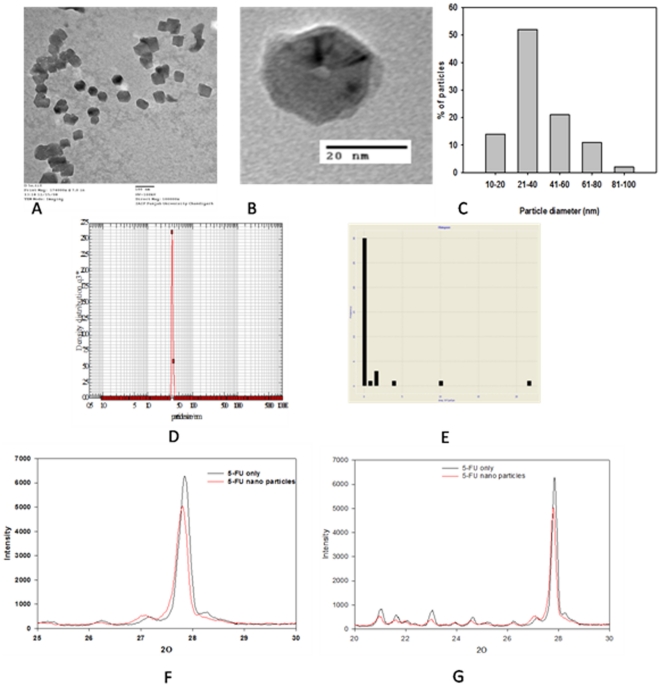
Transmission electron microscopic images of 5-FU nanoparticles. (**A**) Representative TEM image of 5-FU nanoparicles synthesized by incubating 5 ml of 10^−3^ M 5-FU solution with 5 ml of *Aloe vera* extract for 48 h. (**B**) TEM image of the plate like hexagonal 5-FU nano-particles prepared from 5-FU (10^−3^ M) with *Aloe vera* leaf extract (5 ml). HRTEM image shows different section of hexagonal nano-particles of 5-FU. (**C**) Variation in particle size diameter upon incubation of 10^−3^ M 5-FU solution (5 ml) with 5 ml *Aloe vera* extract for 48 h. (**D**) Particle size analysis of 5-FU nano-particles as determined by Nanofox particle analyzer. (**E**) Graph showing size of the nano-particle as obtained by Dynamic Light Scattering studies. (F–G) The X-ray diffraction analysis of 5-FU nano-particles. The X-ray diffraction pattern shows characteristic intense peak at diffraction angle (2θ) of 16°, 19°, 22° and 28°. X-ray diffraction of 5-FU nano-particle was analyzed at diffraction angle (2θ) of (**F**) 20–30 and (**G**) 25–30 respectively.

Figure 2 shows representative transmission electron microscopy (TEM) image of the 5-FU nano-particles synthesized by using *Aloe vera* leaf extract. TEM analysis clearly revealed the formation of hexagonal 5-FU nano-particles. Further studies involving incubation of increasing amount of *Aloe vera* leaf extract with constant amount of drug reveals formation of nanostructures with different shape and surface topology. The size and edge length were found to decrease when higher volume of *Aloe vera* leaf extract was used for synthesis of nano-particles. Incubation with relatively less amount of *Aloe vera* (1 ml *Aloe vera* extract and 5 ml of 10^−3^ M 5-FU solution; final volume of reaction mixture 10 ml) leads to the formation of particles with large and slightly rough edge length (data not shown). However, a different trend was observed when the amount of *Aloe vera* extract was increased from 1 to 5 ml. Nanoparticle synthesis involving larger amount of *Aloe vera* results in the formation of hexagonal nano-particles with sharp edge length (Figure 2A). The bar diagram representing the size distribution of the particles is shown in Figure 2C. A symmetrical edge length analysis of the HRTEM image of a single hexagonal nano-particle noticeably highlights the difference in size and shape of the formed nanostructure (Figure 2B). It can be speculated that incubation of 5-FU solution with relatively large proportion of *Aloe vera* leaf extract leads to the formation of hexagonal structure due to the Multiple Twinned Particles (MTPs). Xia and co-workers [22], [23] have observed that nanostructures evolve from MTPs as a result of anisotropic growth caused by certain shape directing agents. We assume that MTPs play a vital role in the formation of 5-FU hexagonal nanostructure. The formation of isomorphic particles with hexagonal shape indicates that crystallographic plane has highest atomic density. In general, particles with plate morphology grow faster than those with symmetrical shape.

The size of 5-FU nano-particles was further confirmed by nanofox particle analyzer. The size distribution of the particles was observed by digital analysis of image counting of particles. The appearance of a predominant sharp and single peak around ∼40 nm further supported TEM size data (Figure 2D). Size of the 5-FU nanoparticles was also analyzed by dynamic light scattering studies. The distribution of particle diameters showed a main peak at 35 nm along with some insignificant peaks (Figure 2E). The size distribution studies reveal that the particles are more or less mono-disperse with average size of 35±5 nm and further establish that the particle size decreases with an increase in the concentration of *Aloe vera* leaf extract.

Although, it has been the subject of numerous recent investigations [24]–[27], a coherent mechanistic explanation for the evolution of various metal nano-particles prepared via chemical or biomimetic methods has not been delivered yet. A mechanism of nano-particle formation was proposed [8], comprising different steps of particle growth via both coalescence of nuclei and further monomer attachment. Nevertheless, the nature of the ‘directing effect’ is still not clear. One group of researcher claim that it is due to preferred absorption of the capping agent to the surface [28], [29], while others propose that the formation of stacking faults during the first nucleation events is the main factor for asymmetric growth of nano-particles [30], [31].

We found that treatment of *Aloe vera* extract with Proteinase K, DNAse or RNAse does not alter its ability to facilitate formation of 5-FU nanoparticles. This clearly rules out any possible role of nucleic acid or protein based factors present in *Aloe vera* leaf extract in synthesis of 5-FU nano-particles. In an attempt to identify various components of *Aloe vera* leaf extract responsible for particle formation, we dialyzed the extract using a membrane with 3 kD cut off. The components, which regulate induction of nano-particle formation, were found to be less than 3 kD molecular weight as extract when passed through the dialysis bag loses 90% of its activity. We also found that even after complete precipitation with 80% ammonium sulphate the extract was still capable of inducing formation of both gold and 5-FU nano-particles (data not shown). Further, it was found that boiling of extract for 15 min does not eliminate its nano-particle synthesis potential (data not shown). These results are in accordance with the study from other groups suggesting that a water soluble, low molecular weight compound was responsible for synthesis of nano-particles [12].

### Determination of zeta potential of nano-particles

It is well established that size and surface charge of the particles play a major role in its uptake by cells [32]. Further, surface charge, which is generally described as zeta-potential, acquired by a particulate system regulates the half life of a particle *in vitro* as well as *in vivo*. Keeping into consideration the fact that besides nano size, the surface charge also plays important role in regulating the stability of nano particles, we determined zeta potential of 5-FU nano-particles. The zeta potential of 5-FU nanoparticles was found out to be −0.75±0.05 mV, which suggests that particles can withstand biological fluid milieu upon their administration in the host [32].

### Crystalline nature of 5-FU nano-particles

Even though TEM analysis offered direct evidence to confirm formation of 5-FU nano-assemblies, the crystalline structure of nano-assemblies was established by X-ray diffraction study. The anticancer agent, 5-Fluorouracil, is known to exist in two crystalline forms. Form I [33] crystallizes in space group Pī and exhibits an intricate, hydrogen-bonded sheet motif with four crystallographically independent molecules with regions of close F···F contacts. The recently discovered form II (33) (P2_1_/*c*, *Z′* = 1) adopts a hydrogen-bonded ribbon motif, with each molecule forming four NH···O bonds with two adjacent molecules. The layers stack as the (−1 0 2) Miller planes to form the three-dimensional crystal structure [34]. A crystal structure prediction search for lattice-energy minima [34] found form II as the most stable packing arrangement. Recent molecular dynamics simulations [35] suggest that the presence of water enhances the growth of form I, as both carbonyl and amine functional groups remain strongly solvated and, hence, their association into dimers is hindered; while nitromethane facilitates the formation of form II. Therefore we speculate that exclusion of water molecules during nano-assembly formation resulted in 5-FU molecules acquiring form II, which supports our florescence data that dimerisation/polymerisation and collisional quenching interactions between fluorescent moieties during process of nano assembly formation leads to observed fluorescence quenching [36]. As shown in Figure 2F and 2G, X-ray diffraction patterns of nano-assemblies revealed the crystalline nature of nano-particles synthesized from 5-FU with the help of *Aloe vera* extract, and the peaks were much more prominent than the peaks of free 5-FU near the base line at 2θ of 15°–30°. The X-ray diffraction data confirmed that upon nano-particle formation, the structural components (5-FU molecules) maintained their native crystalline structure. Supporting electron microscopic analysis, the XRD data suggest hexagonal structure of 5-FU nano-particles. X-ray diffraction spectrum of pure 5–FU show characteristic intense peaks at diffraction angle with 2θ at 16°, 19°, 22°, and 28°. The peaks establish crystalline nature of the 5-FU nano-particles. X-ray diffraction peaks for free 5-FU were found to have more or less same diffraction pattern except between 2θ of 35°–50°. The XRD studies suggest that nano-assemblies share the same Miller indices with free form of the drug. The most prominent peak (at 2θ of 27.79) showed the same Miller planes (2–1 2) in both cases with d-spacing of 3.16 Å. Further, the XRD data suggest that the structural integrity of parent molecules is retained by 5-FU nanoparticles.

### Atomic Force Microscopic Analysis

**Figure 3:**
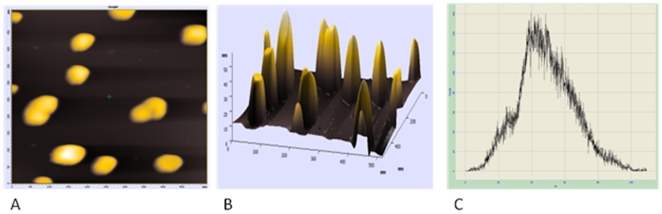
Atomic Force Microscopic study of 5-FU nano-particles. Representative AFM image of 5-FU nano particles synthesized by incubation of 5 ml of 10^−3^ M 5-FU with 5 ml *Aloe vera* extract for 48 hr. (**A**) 2D, (**B**) 3D images of 5-FU nano-particles (**C**) Histogram representation of AFM analysis of 5-FU nano-particles.

Figure 3 shows representative Atomic Force Microscopy (AFM) images of nano drug formulation prepared using 5 ml of *Aloe vera* leaf extract after 48 hr. A number of hexagonal (or similar to hexagonal) nano-particles were observed in the microscopic analysis. Nano-particles can be seen overlapping or in the form of clusters and support TEM data. In concordance with TEM and DLS studies, size of nano-particles was found out to in 30–50 nm range when tracked with 2D image. The height of the 5-FU nano-particle feature is exaggerated for better perception of the fine structures. The AFM also measured height data as visualized with single surface 3D AFM.

### Fourier Transform Infrared Analysis

**Figure 4:**
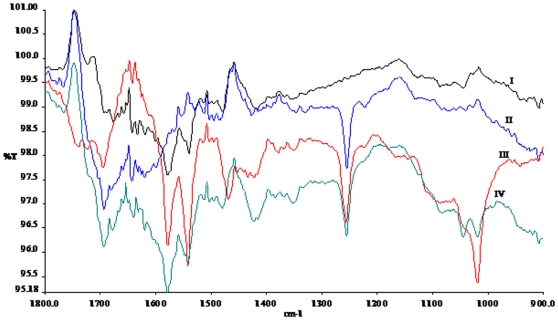
FTIR spectra of 5-FU nano-particles. spectrum 1; *Aloe vera* extract, spectrum II; 5-FU only, spectrum III; 5-FU nano-particles synthesized with 3 ml of *Aloe vera* extract, spectrum IV; 5-FU nano-particles synthesized with 5 ml of *Aloe vera* extract. All the FTIR spectra were recorded after 48 hr of incubation of 5-FU with *Aloe vera* leaf extract.

The 5-FU nano-particles were also characterized by Fourier Transform Infrared (FT-IR) analysis, with the assumption that assembled structures might be ensuing in burial/exposure of various functional groups of parent compound that might lead to changes in corresponding percent transmission intensity [37]. Figure 4 shows the infra red spectra of 5-FU nano-particles, in the region of 900–1800 cm^−1^, obtained under various experimental conditions. FT-IR spectra of leaf extract (spectrum I) shows many intense peaks. Absorption at 1542 cm^−1^ is the characteristic of polyunsubstituted mononuclear ring and a shoulder near 1730 cm^−1^ is due to C = O stretching of carbonyl compounds. This is in accordance with some previous FTIR studies on the same extract [9]. The spectrum of the drug (spectrum II) shows a prominent peak at 1255 cm^−1^, which is due to the stretching of C = O bond of aryl ketone and absorption at 1692 cm^−1^ is characteristic feature of secondary amine in solution as there are two –NH groups in the 5-FU. The spectrum of free drug shows band in the region 1580–1650 cm^−1^ corresponding to the C = N and C = C ring stretched vibration. The absorption band at about 1724 cm^−1^ may be due to the stretching frequency of C = O group. The bands at 1450 cm^−1^ and 1350 cm^−1^ are characteristic for vibration of the substituted pyrimidine compounds [38]. The absorption bands at 1180 cm^−1^ and 1251 cm^−1^ are assigned to the C–O and C–N vibration respectively.

In the spectrum of 5-FU nano-particles (spectrum III and IV) most of the prominent peaks of the parent compound are retained. This suggests that no major change in the chemical structure of 5-FU has occurred upon nano-particle formation. For example, prominent peaks at 1255 cm^−1^, 1692 cm^−1^, 1542 cm^−1^, 1579 and 1730 cm^−1^ are shared between nano-structured and free form of 5-FU. In addition, there are new and intense peaks at 1019 and 1468 cm^−1^, which arise due to exposure or burial of functional group in nano-structure. The appearance of new peak at 1019 cm^−1^ could be because of in plane C–H bending of compound in assembled nano structure. It can also be assumed that the peak at 1019 cm^−1^ is due to formation of S = O group and the S came from the *Aloe vera* extract as there is no S in 5-FU molecule. In another explanation, absorption at 1019 cm^−1^ could be considered as a probe for aryl ether (Ar-O-C) which is probably formed due to complex formation between the aromatic compounds present in the *Aloe vera* extract and C = O moiety of drug. This peak may be due to C–H bending of aromatic ring in plane as well. It is not clear whether absorption at 1468 cm^−1^ is due to C–H bend of δ_s_ CH_2_ of pyrimidine ring originating from the 5-FU nano-particles or due to C-C ring stretching of new complex resulting from the reaction between *Aloe vera* leaf extract and 5-FU. Our FTIR analysis of free form of drug and 5-FU nano-assemblies in the range of 2400–2300 cm^−1^ revealed similar pattern in both the cases (data not shown). We speculate that attainment of particle shape could be the result of either an enzymatic reaction or templating of specific macromolecule that regulates growth kinetics of nano-particle synthesis.

### Release kinetics of drug from 5-FU nanoparticles

**Figure 5:**
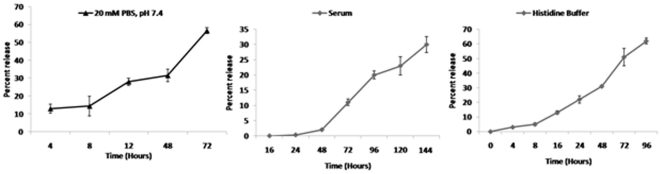
Release profile of 5-FU nano-particles under various conditions. To examine the release kinetics of 5-FU nano-particles, multiple samples of the formulation were dispensed into various micro vials. To each vial, 1.0 ml of release medium (20 mM sterile PBS, serum or histidine buffer) was dispensed. After stipulated time period, the suspension was centrifuged and an aliquot was analyzed for 5-FU content spectrophotometrically as described in Methodology section.

To show that the nano-assemblies can act as a reservoir of 5-FU and facilitate the sustained release of the drug for extended time period, release profile of nano-particles was examined. For this multiple samples of 5-FU nano-particles were dispensed in various micro vials. The release profile showed slow and sustained release of drug over an extended time period. The release kinetic study indicates stability of nano-particles under different pH and temperature conditions. The nano-particles were found to withstand plasma components for a period of more than 120 hr resulting in release of less than 40% of the total drug (Figure 5).

### Cytotoxic potential of 5-FU nano-particles

**Figure 6:**
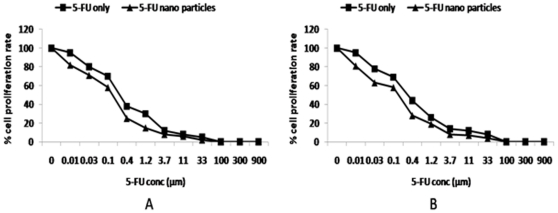
Cytotoxic effect of 5-FU nano-particles. Against (**A**) HT-29 and (**B**) Caco-2 cell lines. MTT assay was used to determine the differential cytotoxicity of 5-FU nano-particles against both cell lines. Cells were dispensed at the density of 5×10^4^ cells per well in U-bottom 96 well plates in triplicate, and treated with increasing concentration of various 5-FU nano formulations. After 48 h of incubation, 20 µl of MTT reagent was added and plate was further incubated for 48 h. It was followed by addition of 100 µl of lysis buffer. The plates were further incubated for 2 hr and absorbance was read at 570 nm.

The cytotoxic potential of formed 5-FU nano-particles against two cell lines, viz. HT-29 and Caco-2 was assessed by determining the number of viable cells surviving after their incubation with drug for stipulated time period using MTT (3-(4,5-dimethylthiazol-2-yl)-5-(3-carboxymethoxyphenyl)-2-(4-sulfophenyl)-2H-tetrazolium) method [39]. The cytotoxicity assay suggests that 5-FU nano-particles exhibit various degree of cytotoxocity against different cancerous cell lines. The cytotoxic effect of 5-FU was prominent against both cell lines, the IC_50_ of free 5-FU was found to be 0.30 and 0.34 µM for HT-29 and Caco-2 cells respectively. Ability of 5-FU nanoparticles to inhibit growth of both the cancer cell lines was found to be time dependant. The IC_50_ of 5-FU nano-particles was 0.21 µM against Caco-2 cells, while the IC_50_ of 5-FU nano-particles against HT-29 cells was 0.25 µM (Figure 6).

### Apoptosis and cell cycle analysis

The anticancer potential of nano-particles was assessed on the basis of expression of various apoptotic factors in treated cells. The pro-apoptotic gene p53 is a key regulator of cell proliferation and cell death [40]. The failure to induce normal expression of functional p53wt leads to unregulated growth of tumors cells. Bax and bcl-2 family proteins also play important role in regulation of apoptosis [41]–[43]. It has been demonstrated that the product of bcl-2 suppresses apoptosis [44], while over-expression of bax gene can inhibit the function of bcl-2 and promote apoptosis [45]. Bax is a p53 target and is known to be transactivated in a number of systems during p53-mediated apoptosis [46].

Normal cell cycle events are tightly regulated by cyclin, cyclin-dependent kinases (CDK), CDK inhibitors (CDKI) and other tumour suppressor genes. The deregulation of cell cycle leads to abnormal proliferation of cells with damaged DNA and evasiveness of apoptosis [47]. Cell cycles are halted at the transition from G1 to S-phase (G1 checkpoint) or from G2 to M-phase (G2 checkpoint) after DNA damage [48]. In the G1 checkpoint, CIP/KIP family including p21^WAF1/CIP1^ and p27^KIP1^, act as inhibitors. Cell cycle block at G2/M phase is known to be associated with inactivation of mitotic Cdks. The observed regulation of p21 upon treatment with 5-FU nanoparticles can be correlated with inhibition of cell cycle kinetics [49]. The Cdk inhibitor p21 binds to cyclin B1-Cdc2 complexes, which blocks the activation of Cdc2 by Cdk-activation kinase thereby prevents the mitotic entry (M phase) [50], [51]. 5-FU is generally believed to induce G1-S-phase arrest of cells, thus halting their progression from G1 to S phase and subsequently paving the way for apoptosis of the cancer cells via a p53-dependent pathway [52]. Other studies have suggested its role in inhibition of cell progression at G2/M phase [53].

**Figure 7:**
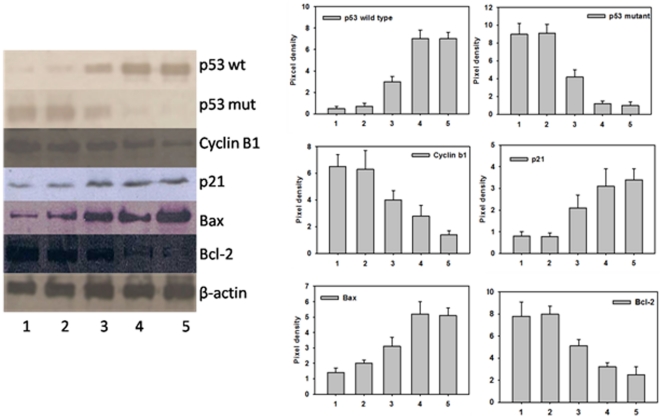
Effect of 5-FU nano-particles on expression of pro/anti apoptotic factors in Caco-2 cell line. Cell lysate was prepared as described in Methodology and analyzed for protein expression using specific antibodies. To quantify equal loading, the membranes were also probed with β-actin antibody. The intensity of bands was quantified using image analysis software on an image gel documentation system. Lane 1, RPMI only; Lane 2, *Aloe vera* leaf extract only; Lane 3, 5-FU only; Lane 4, 5-FU nano-particles prepared by mixing of 5-FU (10^−3^ M) solution with 3 ml of *Aloe vera* extract and Lane 5, 5-FU nano-particles prepared by mixing of 5-FU (10^−3^ M) solution with 5 ml of *Aloe vera* extract.

We extended our study to gain an insight in 5-FU nano-particle mediated modulation of various factors that regulate apoptosis. Western blot analysis was performed to detect level of various cell cycle and apoptotic factors. Incubation of 5-FU nanodrug formulation with cancer cells resulted in enhanced expression of p53wt when compared to free form of the drug (Figure 7). Mutations in p53 gene are commonly found in a majority of malignancies [54]. The p53 protein is known to mediate cell cycle arrest [55] and also acts as a transcription factor that binds to a p53-specific DNA consensus sequence in responsive genes such as p21 [56]. The data of present study suggest that 5-FU nanodrug formulation down regulates the expression of p53mut (two fold decrease) thereby enhancing apoptosis induction in cancer cells. In the control groups (cells treated with *Aloe vera* leaf extract only) the expression profile of regulatory proteins was not significantly affected suggesting that nano-assembled 5-FU is more effective in down regulating p53mut gene expression when compared with free form of the drug. We also analyzed the expression profile of p21 and cyclin B1 of the treated cells and found that nano-drug formulation enhances the p21 protein level comparable with free 5-FU. In the presence of 5-FU nano-formulation, cyclin B1 expression was found to be down regulated by approximately 2.5-fold. Interestingly, 5-FU nanoparticles induce expression of bcl-2 and bax as efficiently as the free form of the drug. The upregulation of bax level in 5-FU nano-particles treated cells (Figure 7) can be correlated with the involvement of mitochondrial (intrinsic) pathway in apoptosis. This results in release of cytochrome c that eventually leading to caspase activation [45].

**Figure 8:**
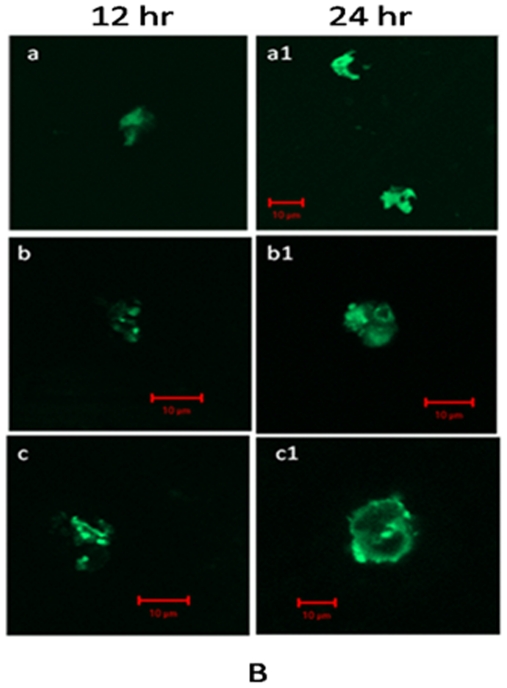
Involvement of caspase-9 in 5-FU mediated apoptosis of Caco-2 cells as revealed by confocal microscopy. Caco-2 cell, were treated with (**a**) RPMI only, (**b**) free 5-FU drug, and (**c**) 5-FU nano-particles for 24 h. After incubation for stipulated time period with various 5-FU preparations, cells were washed and fixed with 4% paraformaldehyde and 0.19% picric acid in PBS (pH 7.4) for 1 h at room temperature (RT). Fixed cells were permeabilized with 0.1% SDS in PBS at RT for 10 min, blocked with 2% FCS, stained with a polyclonal antibody which detects only cleaved 35 kDa caspase-9 (BD, India). The interaction was revealed employing a conjugated goat anti-rabbit IgG-FITC probe (Sigma, India). The cells were then visualized under a fluorescence microscope (excitation at 488 nm emission at 505–530 nm.) scale bar, 10 µm.

Keeping into consideration the fact that caspases are crucial mediators for apoptosis, we evaluated the expression of caspase-9 upon treatment with 5-FU nanoparticles with the help of confocal microscopy. Caspase-9 is the apical caspase in the intrinsic pathway [57]; initiated by cytochrome c released from the mitochondrial intermembrane space into the cytosol [57] that eventually induces apoptosome formation. Expression of caspase-9 was found to be enhanced in a time-dependent manner for 5-FU nanoparticles when compared to the free form of the drug (Figure 8). In case of untreated cells, no increase in the expression of caspase-9 was observed at any time point. Caspase-9 activates the effector caspases-3 and -7, which cleave several other cellular proteins, resulting in the characteristic biochemical and morphological features associated with apoptosis. It seems 5-FU nano form has great potential in induction of caspase-9 and thereby facilitates apoptosis of cells more efficiently when compared to its free form.

The up-regulation of p53 together with down-regulation of bcl-2 expression following 5-FU treatment may partially account for the mechanism involved in 5-FU induced apoptosis in the cells. Of note, both p53- and/or p21- dependent and independent pathways have been previously reported to be involved in 5-FU induced cell cycle arrest and apoptosis in cancer cells; the cell responses varied and the mechanisms may be attributed to the specific cell types involved and the doses of anticancer agents used [53], [58]. Our studies indicate that at low concentration 5-FU nano-particles arrest cells at G2/M phase. This is in accordance with earlier studies that reported effect of free 5-FU on G2/M phase at low concentration, while relatively high concentration of the drug maintained cells in G1 phase [53]. Besides, the nano-particles were also found to upregulate pro-apoptotic factor in a manner similar to free form of the drug. Nevertheless, nano-particles were found to exert their effect more efficiently as equal amount of nano-particles caused approximately three fold increase in p53 wild type and more than two fold decrease in p53 mutant expression when compared to its free form.

## Discussion

**Figure 9:**
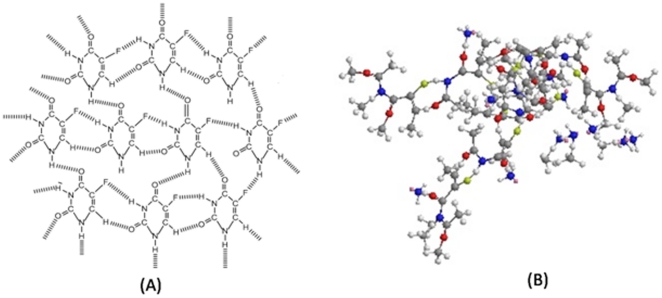
Ball and socket model of 5-FU nano-particles. (**A**) Proposed structure of 5-FU nano-particles, (**B**) Simulated molecular model of 5-FU nanoparticle assembly using ChemDraw software.

Biomimetic synthesis of nano-particles using biological entities is considered as a clean, nontoxic and environment friendly technique. Earlier reports have documented usage of plants such as oat (*Avena sativa*), lemongrass (*Cymbopogon flexuosus*), neem leaf broth (*Azardirachta indica*), amla (*Emblica officinalis*), cinnamon (*Cinnamon camphora*) and *Aloe vera* leaf extract in synthesis of metal (gold, silver, iron and cadmium) based nano-particles. Surprisingly, methods of extending these approaches to organic molecules, such as cancer drug based nano-particles, have not yet been explored. They are highly desirable owing to superiority of nano-particles dosage forms over free form of the drugs. Transformation of parent drug molecules to nanometer size dimension can result in their high uptake across biological membranes. Furthermore, nanosized drug assemblies that are devoid of any pharmaceutical excipients are prerequisite for preparation of more efficacious formulation (Figure 9). Such formulations avoid issues pertaining to biodegradability or biocompatibility *etc* of various carriers (excipients) used in the conventional dosage forms.

In the present study, we have explored the potential of *Aloe vera* leaf extract in synthesis of 5-FU nano-particles. First, we determined the effect of increasing amount of *Aloe vera* extract on synthesis of 5-FU nano-particles. It seems that characteristics features of nano-particles spectra are regulated by shape and size of the particles. Discrete optical transitions have been observed in monodisperse nano-particles system of 5-FU. Both UV and fluorescence spectra show a consistent blue shift with increase in size of the particles. In fact due to their large size, light cannot polarize the nano-particles homogenously and retardation effect leads to the excitation of higher order modes [59]. The synthesis of 5-FU nano-particles was further confirmed by TEM, FT-IR and AFM. All these results correlate well with each other and confirmed the formulation of FU nano-particles.

The nano size of the delivery system is considered of great importance in their uptake by the target cell population. To confirm our hypothesis we performed a set of *in-vitro* experiments employing caco-2 and HT-29 cell line and established that 5-FU nano-particles show better anti-tumor activity when compared to the free form of the drug. Finally, the higher efficacy of 5-FU nanoparticles was established by its ability to markedly down regulate the expression of anti-apoptotic gene when compared to free form of the drug.

### Conclusion

Current research areas in the field of cancer chemotherapy include development of therapeutic delivery systems to allow alternative route of drug administration. Nanomaterials are at the leading edge of the rapidly developing field of nanotechnology. Till now various technologies have been exploited to develop nano-particles. The nano size of the material makes them unique and indispensible in many area of human activity. Biological synthesis of the nano-particles has remained focused on synthesis of metal, however, present study, relying on potential of biomimetic synthesis of organic molecules, opens new vistas in cancer therapy. The *Aloe vera* leaf extract induced development of nanoassemblies of 5-FU that are likely to be more potent when compared to the free form of the drug and need to be explored further for their future use in treatment of cancer in animal models. The exact picture regarding effectiveness of the formulation of 5-FU nano-particles can be obtained if we introduce tiny nanobodies into animal models for cancer therapy. At present we are exploring such developments with the hope that soon it will be possible to introduce novel nano-particle formulations that will not only be more effective but would also be devoid of nano-particle associated putative toxicity constraints.

## Materials and Methods

### Materials

5-Flurouracil, dialysis membrane (MW cut off 3 kD) and bicinchoninic acid (BCA) protein estimation kit were obtained from Sigma Aldrich, USA. Sterile filters of 0.22 µm size were purchased from Millipore. Nitrocellulose membrane, rabbit anti-p53 wild type antibody, rabbit anti-p53 mutant antibody, rabbit anti-cyclin-B1 antibody, and rabbit anti-β-actin antibody were procured from BD Pharmingen and Santa Cruz companies. Caco-2 (ATCC, Number HTB-37) and HT-29 (ATCC, Number HTB-38) cell lines were obtained from ATCC.

### Preparation of *Aloe vera* leaf extract

Thoroughly washed *Aloe vera* leaves (30 g) were finely cut and boiled in 100 ml sterile distilled water as described earlier [12]. The boiled extract was filtered through Whatman filter and the filtrate was stored at −20°C till further use.

### Synthesis of nanodrug formulation using *Aloe vera* leaf extract

Increasing volumes (1–5 ml of 30% w/v solution) of *Aloe vera* leaf extract were added to 5 ml of 10^−3^ M solution of 5-FU in and the volume was made up to 10 ml by deionized water. The mixture was incubated for given time period at room temperature (25°C), and then centrifuged at 20,000 g to pellet the 5-FU nano-particles. The nano-particles were suspended in 1 ml of deionized water and further characterized by various spectrophotometric methods.

### UV and fluorescence spectroscopic analysis

UV spectroscopic analysis of the nano-drug synthesized by *Aloe vera* leaf extract was carried out on a double beam spectrophotometer (Jasco model V-750) operated at a resolution of 1 nm [60]. Fluorescence spectra were recorded with SPEX fluorolog-2 spectroflurometer, corrected for the spectral response of the detection system and smoothed by adjacent averaging.

### Transmission Electron Microscopy

5-FU nano-particles were characterized by TEM studies. The samples for TEM analysis were prepared following protocol as published elsewhere by placing a drop of synthesized nano-particles over gold coated negative grid followed by evaporation of the solvent [61]. TEM analysis was performed on Perkin-Elmer model which was operated at an accelerating voltage of 1000 kV.

### Determination of zeta-potential

Zeta-potential of 5-FU nano-particles was determined by using DTS software (Malvern Instrument Limited, UK) based on M3-PALS technology. Both free as well as nano-particle form of 5-FU were lyophilized in 2 ml microfuge tube and the samples were reconstituted in phosphate buffer, pH 7.4. The dispersion was dispensed into electrophoresis cell to measure the electrophoretic mobility and the data was used to obtain zeta-potential values. The experiments were repeated three times and the average zeta-potential was calculated.

### X-ray diffraction analysis of 5-FU nano-particles

X-ray diffraction pattern of 5-FU nanoparticles was recorded as a function of 2-θ angle. All the diffraction patterns were prepared as step-scans. The 5-FU nano-particles were mounted in lyophilized form to run their step scan, the tube voltage and current were set, and the following parameters entered: starting 2- θ angle, step-size (typically 0.005 degrees), count time per step (typically 0.05–1 second) and ending 2- θ angle. Once started, the goniometer moves through its range, stopping at each step for the allotted time. The X-ray counts at each step were saved to a file on the computer. Once finished, the data were smoothed with a weighted moving average to obtain a diffractogram as displayed in results and discussion. The vertical axis records X-ray intensity and the horizontal axis records angles in degrees 2-theta.

### Atomic Force Microscope (AFM) imaging

A Perkin-Elmer digital instrument multimode scanning probe microscope equipped with a nanoscope controller was used for AFM measurements. Nano-particles formed by co-incubation of 5-FU and *Aloe vera* leaf extract were centrifuged at 20,000 g for 10 min at 4°C and pellet obtained was washed with deionized water to remove any remaining biomass. It was re-suspended in minimal volume of deionized water by brief ultra-sonication and used for drop coating onto a Si (III) disc. Samples were analyzed using contact mode AFM.

### Fourier transform infrared spectroscopy (FTIR) measurement

FTIR measurements of 5-FU nano-particles were carried out on a Perkin-Elmer FTIR Spectrum one spectrophotometer in the diffuse reflectance mode operating at a resolution of 4 cm^−1^ [37]. Each sample was scanned three times to check the authenticity of data. The spectra were taken between 4000 cm^−1^ and 900 cm^−1^ by averaging 128 scans for each spectrum.

### Stability of 5-FU nano-particles in serum, phosphate buffered saline and histidine buffer

To examine the release kinetics of 5-FU nano-particles, multiple samples of the formulation were dispensed into various micro vials. To each vial, 1.0 ml of release medium (20 mM sterile PBS or serum or histidine buffer) was added. The vials were incubated at 37°C. To avoid microbial growth in serum, 0.01% of sodium azide was added. An aliquot (40 µl) of supernatant was removed after centrifugation at 10,000 g for 10 minutes at room temp. The aliquots were replenished with fresh buffer to maintain constant volume of the suspension. HPLC of 5-FU was performed according to published protocol [62] as modified in our laboratory. Briefly, chromatographic separation was carried out using symmetry C_18_, [5 µm column bead sizes (3.9×150 mm)]. Mobile phase consisted of 40 mM phosphate buffer, pH 7.0 with 10% (w/v) potassium hydroxide. Flow rate was kept 1.0 ml/min and detection wavelength was 260 nm. At every time point 20 µl of sample was injected and AUC was recorded. Concentration of sample was calculated from the standard curve of the drug plotted under the same conditions.

### Preparation of cell lysates

The anti-cancer potential of 5-FU nano-particles was assessed on two human cancer cell lines; viz. Caco-2 and HT-29 (human adenocarcinoma colorectal). The cells were cultured as a monolayer in RPMI 1640 at 37°C in CO_2_ incubator (5% CO_2_). Cells were used in the exponential growth phase for the study. After monolayer formation cells were treated with either free or nano-particle formulation of 5-FU. Cellular extracts were prepared following published procedure as standardized in our laboratory [63]. The protein content in the sample lysate was determined using the BCA protein assay kit bovine serum albumin was used as standard.

### MTT assay

The cell lines (Caco-2 cells and HT-29 cells) were maintained in RPMI 1640 culture medium supplemented with 10% heat inactivated fetal calf serum. The cells were plated at a density of 5×10^4^ cells per well in a 96 well plate, and cultured for 72 h at 37°C. The cells were subsequently exposed to various formulations of 5-FU using concentration ranging from 0.01–900 µM. Plates were incubated for 48 h time period and cell proliferation was measured by adding 20 µl MTT dye (5 mg/ml in PBS) per well. Plates were further incubated for another 4 h at 37°C in a humidified chamber containing 5% CO_2_. Formazan crystals formed due to reduction of dye by viable cells in each well were dissolved in 150 µl di-methyl sulfoxide and absorbance at 570 nm was read using SpectraMax M2 plate reader (Molecular devices, USA). Results were expressed as relative absorbance to untreated controls. 5-FU concentration yielding 50% growth inhibition (IC_50_) were calculated using medium effect algorithm and expressed as mean value of three independent experiment.

### Western blot analysis

Western blotting analysis was done to detect various pro and anti apoptotic factor such as p53 wild type, p53 mutant, and cyclin-B1 expressed in the 5-FU treated cell lines [13], [64], [ and 65]. Cells (Caco-2 and HT-29) were lysed after 48 h of treatment with various formulations of 5-FU as described earlier. Protein estimation was done using a BCA kit (Sigma, India) using BSA as standard [66]. Equal amount of sample protein (30 µg) was resolved on 10% SDS-polyacrylamide gel and transferred to nitrocellulose membrane. The membrane was blocked by 5% non-fat dry milk (Bio-rad, USA.) followed by incubation with rabbit anti-p53 wild type antibody, rabbit anti-p53 mutant antibody and rabbit anti-cyclin-B1 antibody. To quantify equal loading, membranes were probed with β-actin antibody (BD Pharmingen). Horseradish-peroxidase-conjugated anti-rabbit and anti-mouse antibodies were used as the secondary antibody. The bands were detected using the ECL (Enhanced chemiluminisence) detection system. The intensity of the bands was quantified using Alpha Image Analysis software on Alpha Image Gel Documentation System.

### Apoptosis analysis

Single cell suspension was prepared by pelleting cells at 2000 *g* for 10 minutes at 4°C. The cell pellet was re-suspended in 50 µl cold PBS and fixed in 2 ml of 70% ice cold ethanol. Cells were washed by sterile PBS followed by treatment with 0.1% Triton X-100 for 5 min. After incubation, cells were centrifuged, resuspended in 1 ml of PBS, mixed with ribonuclease (100 µg/ml) and further incubated at 37°C for 30 min. After pelleting, cells were resuspended in 1 ml of PBS and 50 µg/ml propidium iodide (PI) and incubated for 18 hr at 4°C. Finally, cells were acquired and analyzed on a flow cytometer using ‘Cell Quest 2.0’ software [67].

### Determination of caspase-9 level by confocal microscopy

After treatment with 5-FU nanoparticles for stipulated time period, cells were washed and fixed with 4% paraformaldehyde along with 0.19% picric acid in PBS (pH 7.4) for 1 hr at room temperature (RT). The fixed cells were permeabilized with 0.1% SDS in PBS at RT for 10 min, blocked with 2% FCS, stained with a polyclonal antibody which detects only cleaved 35 kDa caspase-9 (BD, Pharmingen) and revealed with FITC conjugated goat anti-rabbit IgG-FITC conjugated (Sigma) [68]. Confocal microscopy was performed using a Zeiss, 510 Mete equipped with an Argon ion laser mounted on an inverted microscope.

### Statistical analysis

Statistical analysis performed using student's *t*-test. Differences were considered statistically significant with P value <0.05.

## Supporting Information

Figure S1 **Surface Plasmon resonance of gold nanoparticles synthesized using** ***Aloe vera*** **leaf extract.** Visible spectra of gold nanoparticles prepared by incubation of 5 ml of 10^−3^ M of HAuCl_4_ with increasing concentration of *Aloe vera* leaf extract for 24 h. The peak intensity of characteristic surface plasmon resonance (SPR) band of gold nanoparticles increases with increasing amount of *Aloe vera* leaf extract as mentioned in graph. The inset shows color photo of various nanoparticle solution formed after 24 h of reaction of *Aloe vera* extract with 10^−3^ M of HAuCl_4_. The labels of various samples in inset correspond to (**a**) 1 ml of HAuCl_4_ only, (**b**) HAuCl_4_ incubated with 1 ml *of Aloe vera* extract (**c**) HAuCl_4_ incubated with 3 ml of *Aloe vera* extract (**d**) HAuCl_4_ incubated with 5 ml of *Aloe vera* extract.
(TIF)
