# Supplementary figures and images for: Aloe vera Induced Biomimetic Assemblage of Nucleobase into Nanosized Particles
Source: PLoS One. 2012 Mar 5;7(3):e32049. doi: 10.1371/journal.pone.0032049 (PMC3293877; doi:10.1371/journal.pone.0032049)

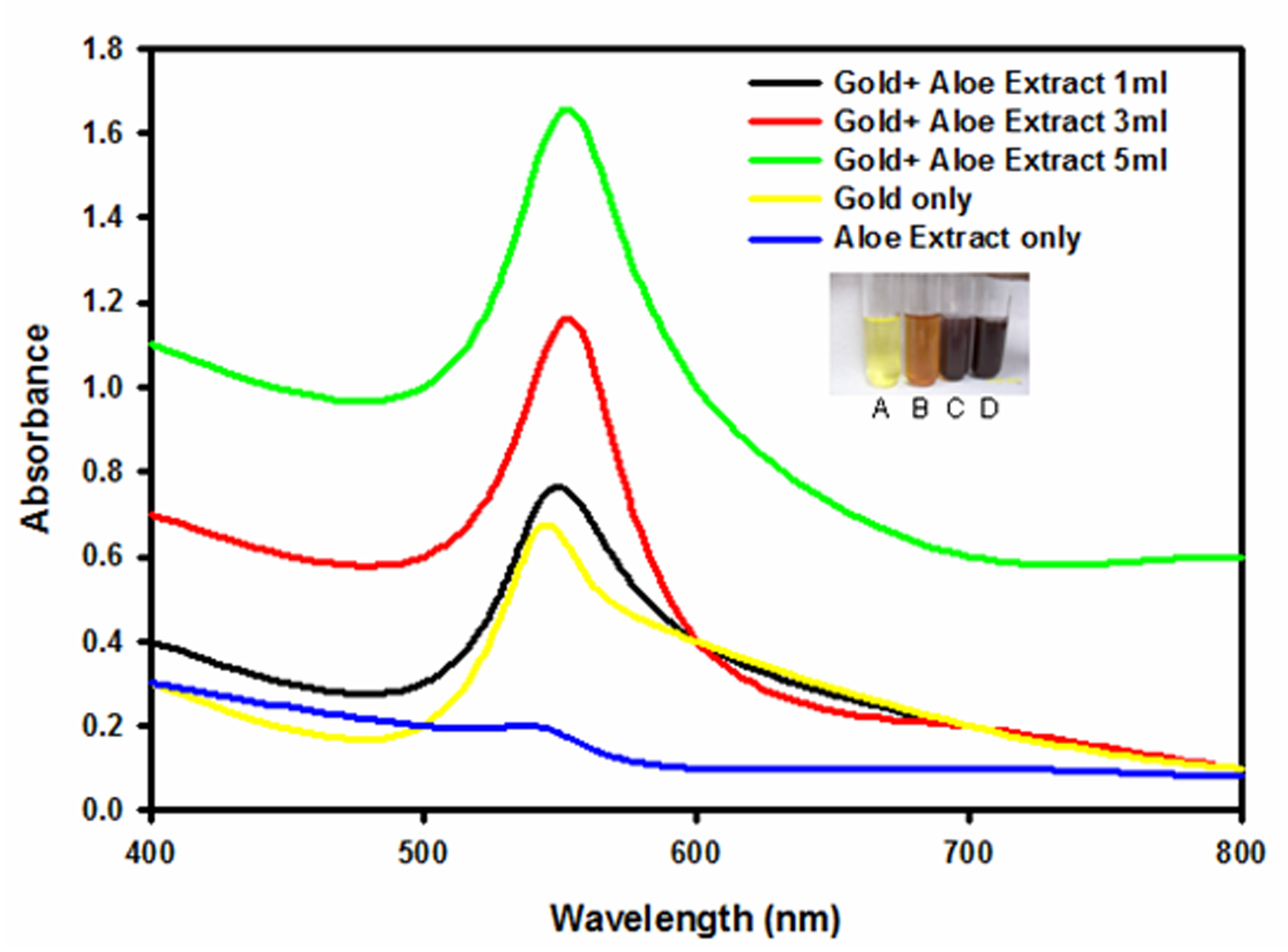

Supplement: Figure S1 — Surface Plasmon resonance of gold nanoparticles synthesized using Aloe vera leaf extract. Visible spectra of gold nanoparticles prepared by incubation of 5 ml of 10−3 M of HAuCl4 with increasing concentration of Aloe vera leaf extract for 24 h. The peak intensity of characteristic surface plasmon resonance (SPR) band of gold nanoparticles increases with increasing amount of Aloe vera leaf extract as mentioned in graph. The inset shows color photo of various nanoparticle solution formed after 24 h of reaction of Aloe vera extract with 10−3 M of HAuCl4. The labels of various samples in inset correspond to (a) 1 ml of HAuCl4 only, (b) HAuCl4 incubated with 1 ml of Aloe vera extract (c) HAuCl4 incubated with 3 ml of Aloe vera extract (d) HAuCl4 incubated with 5 ml of Aloe vera extract. (TIF) [file pone.0032049.s001.tif]
